# Sugar-induced cell death is temperature dependent and conserved in *Saccharomyces cerevisiae* and *Candida* species

**DOI:** 10.1128/spectrum.01568-25

**Published:** 2025-10-13

**Authors:** Raveena Parbhudayal, Hai-Ping Cheng

**Affiliations:** 1 Department of Biological Sciences, Lehman College, The City University of New York, Bronx, New York, USA; 2 The Graduate Center, City University of New York, New York, New York, USA; Agroscope, Nyon, Switzerland

**Keywords:** sugar-induced cell death, yeast programmed cell death, glucose signaling, metabolic flexibility

## Abstract

Yeast sugar-induced cell death (SICD) is a phenomenon whereby cells lose viability in the presence of glucose and the absence of other nutrients to support their growth. Both stationary- and exponential-phase cells of *Saccharomyces cerevisiae* undergo SICD. The majority of studies on SICD are focused on *S. cerevisiae*; however, since glucose is a universal carbon source for yeast, we hypothesized that a similar response may be seen in other species of yeast. In this study, we sought to conduct a comparative analysis of SICD in *S. cerevisiae* and *Candida* spp. We first established that SICD is prevalent in *S. cerevisiae* and *Candida* spp.; however, this phenomenon is highly dependent on temperature, which may explain why this phenotype was perhaps missed previously in *C. albicans*. We found that glucose is a universal inducer of cell death in all tested strains, but other carbon sources may also lead to cell death to a lesser extent. Additionally, cells transferred to a glucose-only solution induce a dramatic decline in extracellular pH, which may not be directly associated with cell death. Together, our data suggest that SICD is a fundamental and conserved mechanism in yeast.

**IMPORTANCE:**

Since the discovery of the yeast metacaspase, *YCA1*, research on cell death and aging in *Saccharomyces cerevisiae* has expanded significantly. Increasing evidence demonstrates similarities between yeast and mammalian cell death. A less discussed type of cell death is sugar-induced cell death (SICD). SICD is a phenomenon observed in *S. cerevisiae*, whereby cells transferred to water-only remain viable for many days; however, when transferred to glucose-only solutions, there is a rapid loss of viability. Studies on SICD in yeast have been focusing mostly on *S. cerevisiae*. In this study, we expand the systematic characterization of SICD in *Saccharomyces* to pathogenic *Candida* spp. The extension of SICD in pathogenic yeast raises the possibility of this mechanism being of potential interest in therapeutic development.

## INTRODUCTION

Sugar-induced cell death (SICD) in yeast is a phenomenon first described by David Granot, whereby cells rapidly lose viability when transferred to glucose-only solutions (1–3). Additionally, supplementation of nitrogen-containing compounds protects against SICD, supporting the idea that SICD may be a result of an imbalance in the nitrogen and carbon available for the cells to support growth (4). SICD can be induced by both fermentable and non-fermentable carbon sources (2) and was shown to be dependent on cellular entry and phosphorylation of the sugar (1, 2, 5). When stationary-phase cells of *S. cerevisiae* are transferred to a glucose-only solution, cell death is accompanied by DNA and RNA degradation, membrane blebbing, membrane fragmentation, vesicle formation, and nuclear fragmentation—all hallmarks of mammalian apoptosis (3). However, cell death is independent of the only identified yeast metacaspase, *YCA1* (6). On the other hand, when exponential-phase cells of *S. cerevisiae* are transferred to glucose-only solutions, the cell death phenotype resembles that of primary necrosis. Cell death here is induced within 15 minutes, with evidence of membrane damage, swollen nuclei, chromatin release into the cytoplasm, an absence of phosphatidylserine externalization, and independence from *de novo* protein synthesis (7–9).

In both stationary- and exponential-phase cells of *S. cerevisiae*, SICD is associated with an increase in intracellular ROS levels. Treatment with antioxidants reduces ROS levels and rescues *S. cerevisiae* from cell death (3, 8–11). SICD also results in rapid acidification of the extracellular environment (9, 12). Under neutral pH, SICD is prevented with a corresponding decrease in intracellular ROS accumulation (9). Additionally, glucose causes a dramatic increase in membrane potential, which is diminished by supplementation of KCl or neutralizing pH. This led to a proposed mechanism for SICD, whereby glucose falsely activates H^+^-ATPases in the absence of external K^+^, leading to dielectric breakdown of the plasma membrane and causing small molecules, including K^+^ ions, to leak out of the cells. Cells undergoing SICD also experience enhanced O_2_ consumption, suggesting disruption of the Crabtree effect. Accordingly, downregulation of respiration protects against cell death (11, 13).

SICD was described in *S. cerevisiae* (1–6, 8–10, 14–16) and was not detected in *C. albicans* (17). Instead, it was reported that *C. albicans* undergoes cell death in response to N-acetylglucosamine (Glc-NAc), whereby cells exposed to Glc-NAc experience rapid cell death due to increased accumulation of ROS. The cell death in *C. albicans* was associated with markers of apoptosis, followed by necrosis (17). This study concluded that Glc-NAc may act as a false signal for high-nutrient conditions, leading to increased cellular metabolism, preventing exit from the cell cycle, and eventually resulting in cell death (17).

SICD studies have been reported from many labs; however, the method of induction is yet to be standardized for uniform and reliable responses, as the molecular and genetic mechanisms are being elucidated. In this study, we aim to conduct a comparative analysis of SICD in *S. cerevisiae* and *Candida* spp. to assess the prevalence of SICD in yeast and gain insights into the evolutionary importance of SICD in yeast. Concurrently, we investigate the importance of environmental factors, specifically temperature and pH, on SICD.

## MATERIALS AND METHODS

### Strains and media

The strains used in the study are shown in Table 1.

**TABLE 1:** Yeast strains used in this study

Species	Strain	Genotype	Source
*Saccharomyces cerevisiae*	X2180-1A	*MATa SUC2 mal mel gal2 CUP1*	Dr. Peter Lipke
	X2180-1α	*MAT*α *SUC2 mal mel gal2 CUP1*	Dr. Peter Lipke
	S288c/ATCC 204508	*MAT*α *SUC2 mal mel gal2 CUP1 flo1 flo8-1 hap1*	ATCC
	S288c-PDA^*a*^	S288c isolated on potato dextrose agar	Internal Stock
	BY4739	*MAT*α *leu2*Δ*0 lys2*Δ*0 ura3Δ0*	Horizon Discovery
	BY4741	*MATa, his3*Δ*1, leu2*Δ*, met15*Δ*0, ura3*Δ*0*	Horizon Discovery
*Candida albicans*	ATCC 18804	–^*b*^	ATCC
	SN250	–	Dr. Jason Rauceo
	SC5314	Clinical isolate	Dr. Jason Rauceo
	DAY286	–	Dr. Jason Rauceo
*C. glabrata* (*Nakaseomyces glabratus*)	–	–	Dr. Jason Rauceo
*Candida dubliniensis*	–	–	Dr. Jason Rauceo
*Candida parapsilosis*	–	–	Dr. Jason Rauceo
*Candida auris*	ATCC MYA5001	–	ATCC

^*a*^ Sequence deposited into NCBI as *Saccharomyces cerevisiae* S288c-PDA, accession number JBPPHI010000000.

^*b*^ – indicates that the information for the strains is unavailable.

The medium used in this study is YPD, which consists of 1% yeast extract (Difco), 2% peptone (Sigma), and 2% dextrose (Sigma).

Stock solutions of glucose (Sigma) and sorbitol (USB Corporation) were prepared at 40% concentration, while sucrose, fructose, arabinose, mannitol, galactose, succinate, pyruvate, and glycerol were prepared at 20% concentrations, and ethanol was used from a 90% stock solution. All listed carbon sources were purchased from Sigma.

### SICD assays

SICD was induced in exponential- and stationary-phase cells according to previous studies (3, 8) with minor modifications. Yeast cells were cultured to exponential phase (overnight, between 16 and 18 hours) or stationary phase (48 hours) in YPD medium at 30°C with shaking at 200 rpm. All starting cultures were inoculated to an OD_600_ of 0.01. The cells were washed twice in autoclaved water and then used to inoculate 20 mL of 2% sorbitol (or water) or 2% sorbitol + 2% glucose (unless otherwise stated) in 125 mL flasks to OD_600_ of 1.3. The flasks were incubated at 37°C with shaking at 200 rpm. Sucrose, fructose, galactose, arabinose, and mannitol were also assessed for their ability to induce SICD at a 2% concentration in water, while succinate, pyruvate, glycerol, and ethanol were assessed at a 4% concentration in water.

The number of viable cells was determined by serial dilutions in YPD medium using 96-well plates, and the cells were plated on YPD agar plates. The plates were incubated at 30°C for 48 hours, and then the number of colony-forming units (CFUs) was counted.

Exponential-phase *C. albicans* SN250, ATCC 18804, DAY286, and SC5314 were prepared by transferring 2 mL of overnight cultures to 25 mL of fresh YPD medium and subculturing them at 30°C for 4–6 hours with shaking at 200 rpm.

The average OD_600_ of exponential-phase *S. cerevisiae* and *Candida* spp. was less than 9.0 and 15, respectively. The OD_600_ of stationary-phase *S. cerevisiae* and *Candida* spp. ranged between 8–15 and 25–50, respectively.

### Growth assays

To determine the effect of carbon sources on growth, 2% glucose, fructose, sucrose, galactose, arabinose, mannitol, lactose, and sorbitol, and 4% ethanol, pyruvate, succinate, and glycerol were supplemented to YP-only (yeast extract and peptone only) to a total volume of 5 mL in test tubes. The tubes were inoculated to an OD_600_ of 0.01 with respective strains, and they were incubated at 30°C with shaking at 200 rpm. Growth was determined at 48 hours by measuring the OD at 600 nm using the BioRad SmartSpec 3000 UV-Vis Spectrophotometer.

### pH measurement

Either water or 2% glucose was inoculated to OD_600_ of 1.3 with respective strains of *Candida* using stationary-phase cells and incubated at 37°C with shaking at 200 rpm. To measure pH, 500 µL of culture was transferred to an Eppendorf tube and centrifuged at 13, 000 rpm for five minutes at room temperature. The supernatant was transferred to a clean Eppendorf tube, and pH was determined using a Dual pH Meter (Spectrum Technology, Inc.).

### Construction of phylogenetic tree

To construct the phylogenetic tree, 18S rRNA sequence of each strain was obtained from NCBI on 25 October 2024. The accession numbers were NG_063315.1 (*S. cerevisiae*), NG_063242.1 (*C. glabrata*), NG_070791.1 (*C. albicans*), NG_073563.1 (*C. auris*), NG_073564.1 (*C. parapsilosis*), and NG_062654.1 (*C. dubliniensis*). The sequences were copied into Clustal Omega (https://www.ebi.ac.uk/jdispatcher/msa/clustalo) for multiple sequence analysis (MSA), and a phylogenetic tree was generated by Clustal Omega based on the MSA.

### Statistics

Data in the line graphs are presented as mean ± standard deviation, while data in the bar charts are shown as mean with range.

## RESULTS

### SICD is more rapid in exponential-phase cells of *S. cerevisiae* than stationary-phase cells

**Fig 1:**
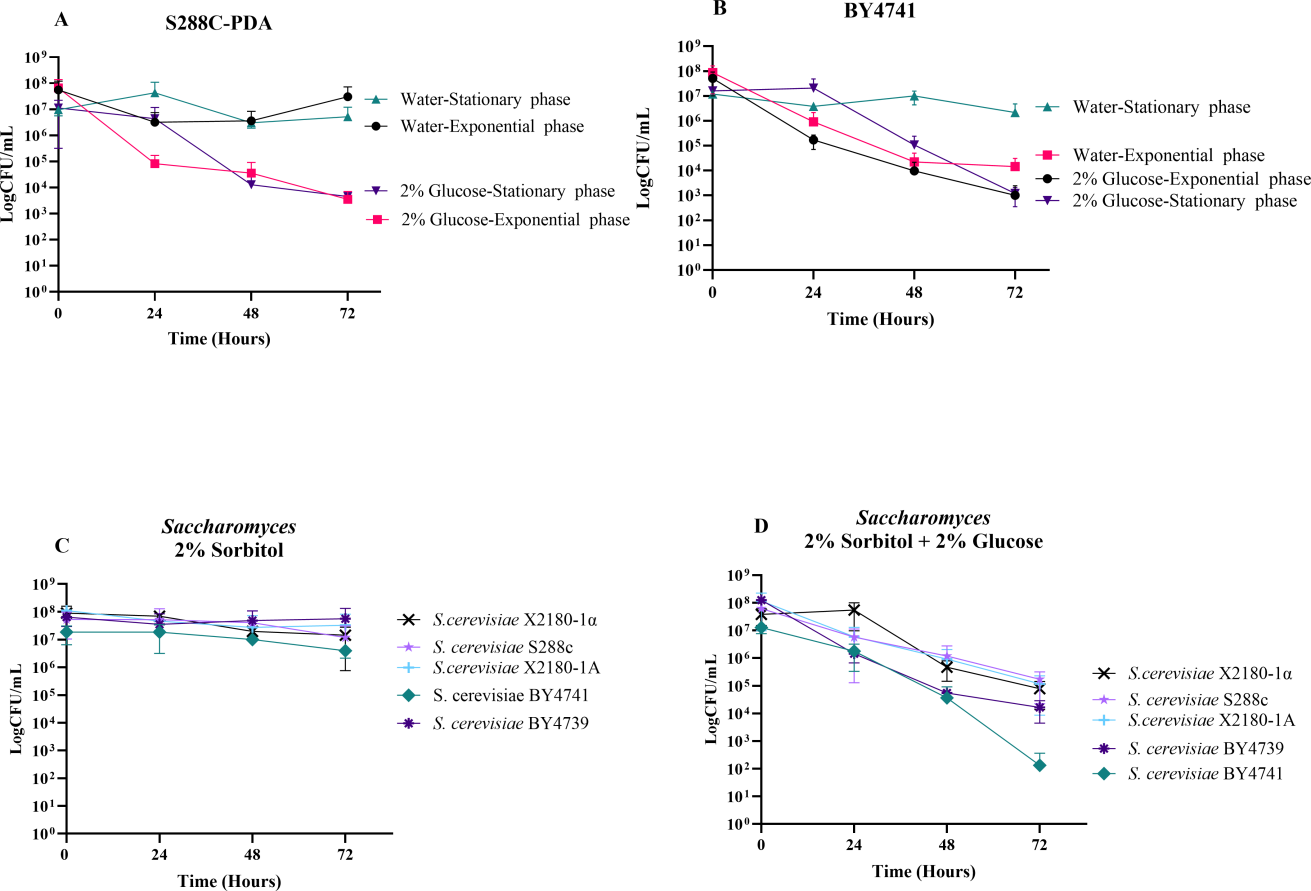
Characterization of SICD in *S. cerevisiae*. The survival of different strains of *S. cerevisiae* was determined by monitoring CFU over 72 hours. Exponential- and stationary-phase cells of *S. cerevisiae* S288c-PDA (**A**) and BY4741 (**B**) were transferred from YPD medium to water-only or 2% glucose solutions and incubated at 37°C with shaking at 200 rpm. Survival was determined at 24-hour intervals. SICD was also investigated in five other strains of *S. cerevisiae* by inoculating exponential-phase cells in either 2% sorbitol (**C**) or 2% sorbitol + 2% glucose (**D**). Survival was determined at 24-hour intervals by CFU assays, and in all cases, the cells were plated in YPD agar plates and incubated at 30°C for 48 hours. The data presented are mean ± standard deviation.

Independent studies have shown that SICD occurs in exponential-phase and stationary-phase cells; however, it appears to be more rapid in exponential-phase cells (3, 8). To study SICD systematically, viability of stationary- and exponential-phase cells of *S. cerevisiae* S288c-PDA and BY4741 was followed for three days (Fig. 1A and B). The cells were grown in YPD medium, washed, and transferred to either water or 2% glucose solutions, and cell survival was determined at 24-hour intervals.

Our results showed that both exponential- and stationary-phase cells of S288c-PDA remained viable in water for 72 hours, while incubation in 2% glucose resulted in a 4-log reduction in viability (Fig. 1A). When BY4741 was examined, viability of stationary-phase cells remained unchanged in water, while viability of exponential-phase cells declined by 4 logs (Fig. 1B). Glucose resulted in a 5-log reduction in viability for stationary and exponential-phase cells of BY4741 (Fig. 1B). In addition, the viability of exponential-phase cells of both strains appeared to decrease more rapidly within the first 24 hours than that of the stationary-phase cells when exposed to glucose-only. Our data confirm that SICD occurs in exponential and stationary-phase cells, and exponential-phase cells are more sensitive to SICD.

The decline in viability of exponential-phase BY4741 (Fig. 1B) in water-only is likely due to osmotic stress, as other studies have used sorbitol as an osmoprotectant (3). However, when 2% sorbitol was supplemented, the protective effect on exponential-phase cells was negligible in this strain (data not shown). For all of our future experiments using BY4741, stationary phase cells were used, and 2% of sorbitol was used instead of water-only, unless otherwise stated.

### SICD is observed in all tested lab strains of *S. cerevisiae* and in both mating types

SICD was previously reported in a few strains of *S. cerevisiae*. We suspected that other strains may also exhibit this phenotype. To test this hypothesis, we collected common lab strains of *S. cerevisiae*, including both mating types, and assessed SICD in exponential-phase cells. When transferred to 2% sorbitol, the viability of all strains remained unchanged at 72 hours (Fig. 1C); however, transferring to 2% glucose resulted in a nearly 5-log and 3-log decrease in viability of BY4741 and BY4739, respectively, and a 2-log decrease in viability of X2180-1A, X2180-1α, and S288c (Fig. 1D). It should be noted that a decrease in viability occurred in both mating types of the X2180 strain; however, viability of X2180-1α began decreasing after 24 hours. Nevertheless, both mating types resulted in the same extent of cell death by 72 hours. This data suggest that SICD may be prevalent in *S. cerevisiae,* and it is independent of mating type.

### *Candida albicans* undergoes SICD at 37°C

**Fig 2:**
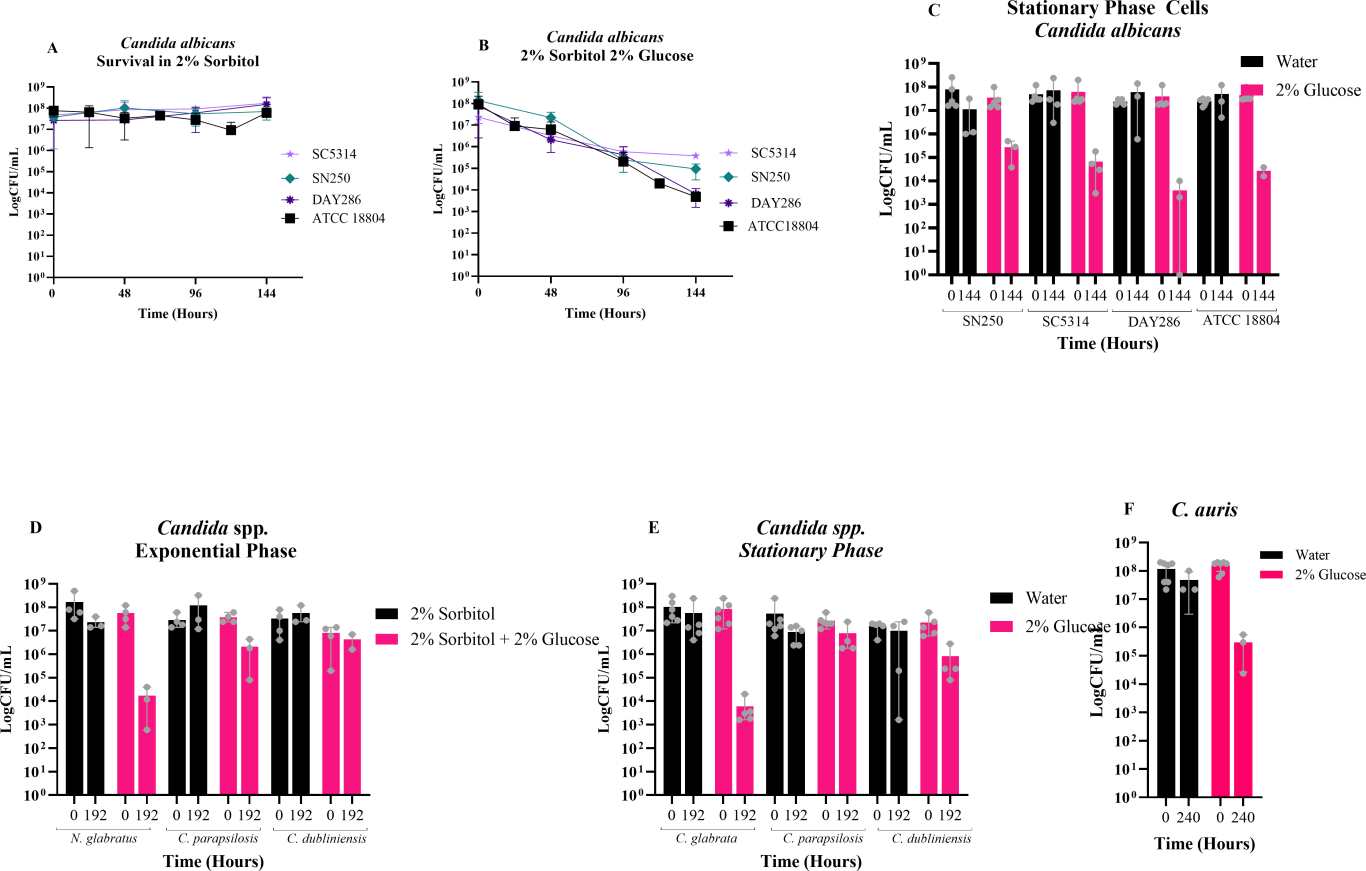
Determination of SICD in *Candida*. Exponential-phase cells of *C. albicans* ATCC 18804, SN250, DAY286, and SC5314 were transferred to 2% sorbitol (**A**) or 2% sorbitol + 2% glucose (**B**), and CFU assays were done at 48-hour intervals to monitor viability. Stationary-phase cells of *C. albicans* ATCC18804, SN250, DAY286, and SC5314 (**C**) were tested by transferring stationary-phase cells from YPD medium to either water or 2% glucose. Exponential-phase (**D**) and stationary-phase (**E**) cells of *C. dubliniensis*, *C. glabrata*, and *C. parapsilosis* were tested by transferring exponential- or stationary-phase cells to either 2% sorbitol and 2% sorbitol + 2% glucose or water and 2% glucose, respectively. Stationary-phase cells (**F**) of *C. auris* ATCC MYA5001 were also tested by transferring cells to either water or 2% glucose solutions. CFU assays were done at 48-hour intervals to monitor viability. The data presented in panels A and B are mean ± standard deviation. The data presented in panels C to F are mean with range. Each dot represents data collected from independent replicates.

Since SICD was observed in all the tested strains of *S. cerevisiae*, the next task was to determine if *C. albicans,* the most common pathogenic yeast, would also undergo SICD. It was previously reported that SICD does not occur in *C. albicans*; however, the experiment was done at 30°C (17). Our current protocol for assessing SICD is performed at 37°C, as recommended by David Granot (1, 3). To test for SICD, exponential-phase *C. albicans* ATCC 18804, SN250, DAY286, and SC5315 were transferred to either sorbitol or sorbitol with glucose and incubated at 37°C. As shown in Fig. 2A, the viability of all strains remained unchanged in sorbitol for up to 144 hours; however, in the presence of glucose, there was a 4-log decrease in viability of ATCC 18804 and DAY286, 3-log decrease for SN250, and 1-log decrease with SC5314 (Fig. 2B). We initially observed that SICD in *C. albicans* occurred slower than that of *S. cerevisiae*; hence, sampling was done at 48-hour intervals.

Stationary-phase cells of *C. albicans* were also evaluated using the four strains, ATCC 18804, SN250, SC5314, and DAY286. As shown in Fig. 2C, the viability of all strains remained similar in water at 144 hours, except for SN250, where there was a small reduction in viability. It is noteworthy that at 96 hours, viability was similar to that at 0 hours for SN250. In the presence of glucose, the viability of SN250 declined by 2 logs, while the viability of SC5314 and ATCC 18804 declined by 3 logs, and the viability of DAY286 declined by 4 logs. These results contradict previous studies showing that SICD does not occur in *C. albicans*.

### SICD occurs in other *Candida* species

Since SICD occurred in all tested *C. albicans* strains, the next question was whether this occurs in other species of pathogenic *Candida*. To determine this, we tested *C. dubliniensis*, *C. glabrata*, and *C. parapsilosis* and the newly emerged *C. auris*. As shown in Fig. 2D, when exponential-phase cells were transferred to 2% sorbitol, the viability at 192 hours was similar to 0 hours. However, in the presence of 2% glucose, there was a 4-log reduction in viability of *C. glabrata* and a 1-log reduction in viability of *C. parapsilosis*. Interestingly, the viability of *C. dubliniensis* remained unchanged in the presence of glucose.

When stationary-phase cells were tested (Fig. 2E), the viability of *C. glabrata* declined by 4 logs in glucose while remaining unchanged in water. Viability of *C. dubliniensis* declined by 1 log in glucose; however, in some replicates, viability also declined in water-only. This is not surprising, as *C. dubliniensis* was previously shown to be sensitive to osmotic stress (18). On the other hand, the viability of *C. parapsilosis* remained relatively similar in water and glucose. Taken together, our data show that SICD can occur in both exponential- and stationary-phase cells of *C. glabrata*, in exponential-phase cells of *C. parapsilosis*, and that SICD does not occur in *C. dubliniensis*.

The emergent multi-drug-resistant fungal pathogen, *Candida auris*, was tested for its ability to undergo SICD using the strain *C. auris* ATCC MYA5001. Exponential-phase cells were overly sensitive to osmotic stress at 37°C and utilized sorbitol. Therefore, the method used to test for SICD in exponential-phase cells did not apply to this strain. However, when stationary-phase cells were tested, cell viability remained unchanged in water for 240 hours, but glucose resulted in a 2-log reduction in viability at 240 hours (Fig. 2F).

### SICD in *S. cerevisiae* is dependent on temperature

**Fig 3:**
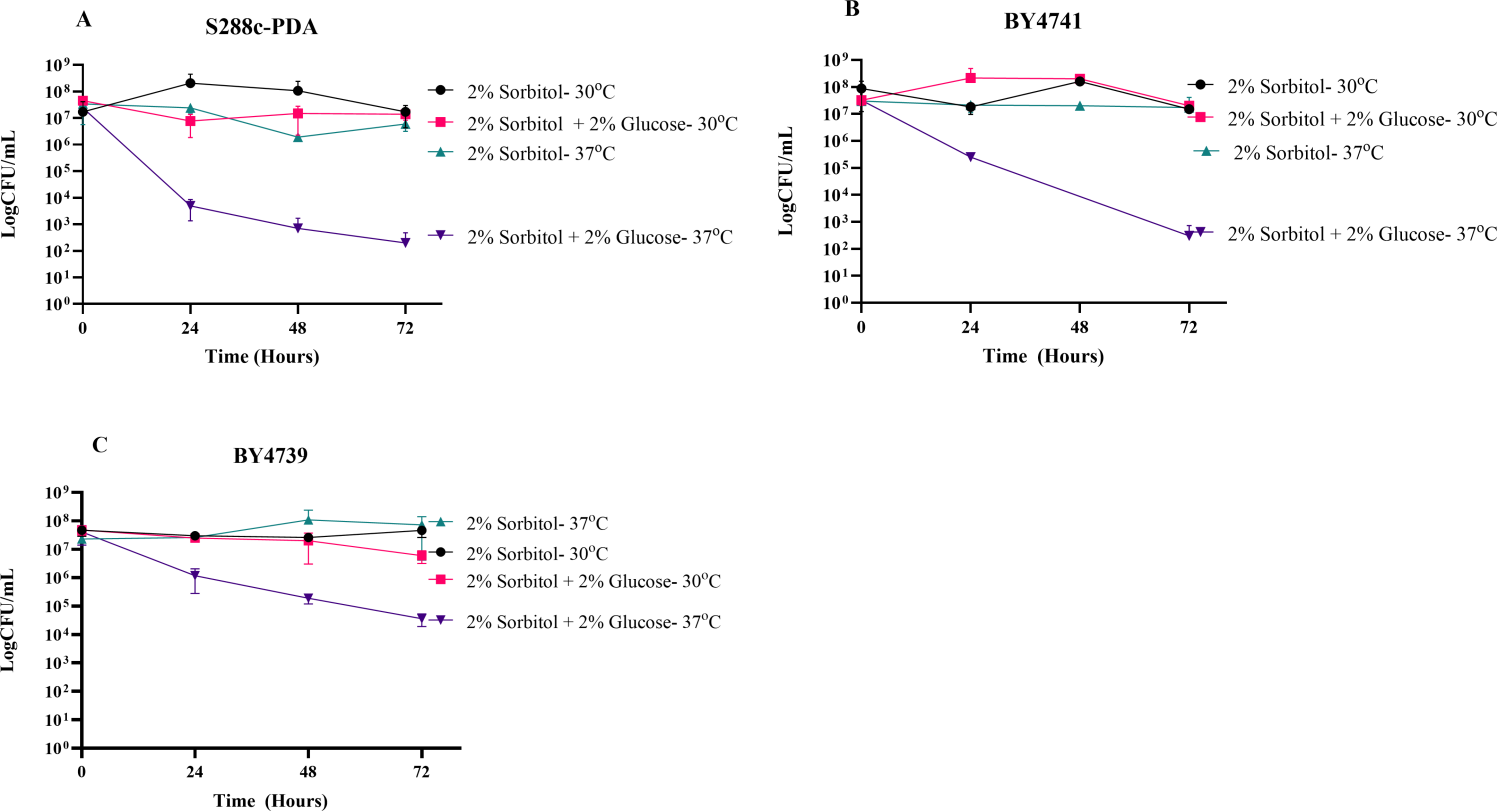
The effect of temperature on SICD in *S. cerevisiae*. Exponential-phase cells of S288c-PDA (**A**) and BY4739 (**C**) and stationary-phase BY4741 (**B**) were transferred to either 2% sorbitol or 2% sorbitol + 2% glucose, and they were incubated at 30°C and 37°C with shaking. CFU was determined at 24-hour intervals for up to 72 hours. The data presented is mean ± standard deviation.

While the implications of temperature on SICD remain unclear, the literature indicates that SICD is enhanced at 37°C (1, 5); however, published comparative analysis of SICD at 30°C and 37°C is not available. Here, the effect of temperature on SICD was investigated using exponential-phase cells of *S. cerevisiae* S288c-PDA and BY4739 and stationary-phase *S. cerevisiae* BY4741. As shown in Fig. 3A through C, the viability of all strains at 30°C in 2% glucose remained unchanged at 72 hours; however, at 37°C, glucose resulted in a 3-log reduction of viability. These findings suggest that temperature plays a critical role in the induction of SICD in *S. cerevisiae*.

### SICD in *C. albicans* is temperature-dependent

**Fig 4:**
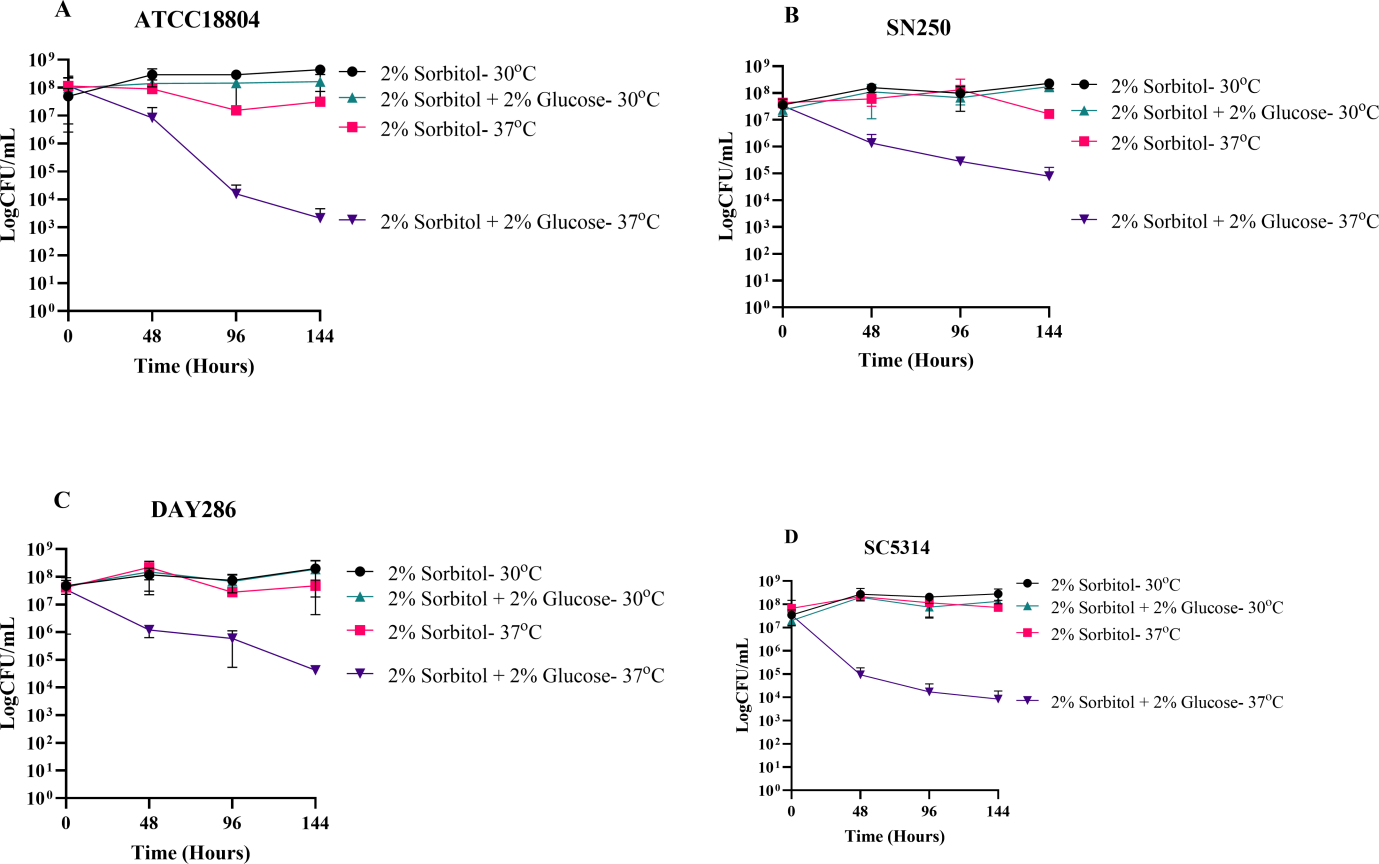
The effect of temperature on SICD in *C. albicans*. *C. albicans* ATCC 18804 (A), SN250 (B), DAY286 (**C**), and (**D**) SC534 were cultured to the exponential phase and then transferred to either 2% sorbitol or 2% sorbitol + 2% glucose. They were incubated at 30°C or 37°C for 144 hours. CFU assays were done to determine the change in viability at 48-hour intervals. The data presented are mean ± standard deviation.

To confirm if temperature plays a role in SICD in *C. albicans*, we monitored viability of exponential-phase *C. albicans* ATCC 18804, SN250, DAY286, and SC5314 at 30°C and 37°C. We found that at 144 hours, viability of all the strains remained unchanged in 2% sorbitol at 37°C and 30°C and in glucose at 30°C. However, the viability of all strains in glucose at 37°C was reduced by at least 2 logs (Fig. 4A through D). These results suggest that the induction of SICD in *C. albicans* is temperature-dependent and explains why this phenomenon was previously misrepresented in the literature.

### Metabolism and cell death may be directly related in *S. cerevisiae*

**Fig 5:**
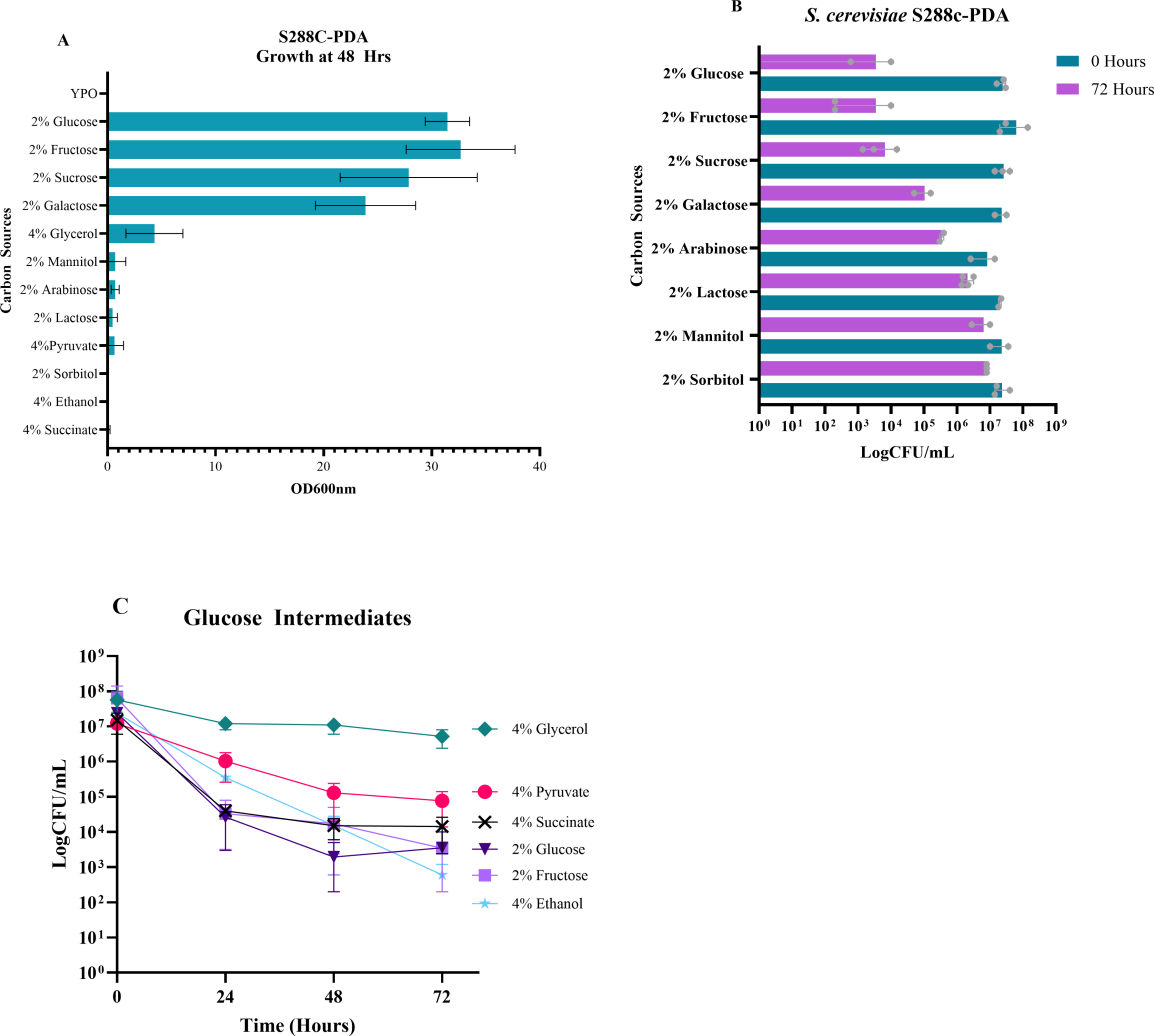
The effect of other carbon sources on survival. The effect of glucose, fructose, sucrose, galactose, glycerol, mannitol, lactose, arabinose, pyruvate, succinate, ethanol, and sorbitol as the sole carbon source for growth was investigated in YP-only as the above-stated concentrations using *S. cerevisiae* S288c-PDA (**A**). OD_600_ was measured at 48 hours to detect growth. All carbon sources were then tested for their ability to induce SICD using exponential-phase cells of *S. cerevisiae* S288c-PDA. The cells were incubated at 37°C, and viability was followed for 72 hours (**B**). The effect of the metabolism intermediates on SICD was also assessed using 4% of each intermediate then tracking viability for 72 hours (**C**). The data presented in panels A and C are mean ± standard deviation, while the data presented in panel B are mean with range. Each dot represented data from independent replicates.

It was previously shown that fermentable and non-fermentable carbon sources can induce SICD in *S. cerevisiae* (2); however, we questioned whether there is a relationship between metabolism and cell death. Therefore, a range of carbon sources were screened for their ability to support growth to identify metabolizable and non-metabolizable carbon sources for S288c-PDA. This was determined by inoculating *S. cerevisiae* S288c-PDA in YP-only medium supplemented with 2% of either glucose, fructose, sucrose, galactose, arabinose, lactose, mannitol, or sorbitol as the sole carbon source. An increase in OD_600_ above that observed in YP-only after 48 hours of incubation indicates the carbon source supports growth. As shown in Fig. 5A, glucose, fructose, sucrose, and galactose supported the growth of *S. cerevisiae* S288c-PDA. However, mannitol, lactose, arabinose, and sorbitol did not. It is noteworthy that growth in galactose was initiated after 24 hours.

Both metabolized and non-metabolized carbon sources were then tested for their ability to induce SICD in *S. cerevisiae* S288c-PDA (Fig. 5B). The results showed that glucose, fructose, and sucrose caused a 3-log reduction in viability by 72 hours. Galactose resulted in a 2-log reduction in viability, while arabinose and lactose resulted in a 1-log reduction. Sorbitol and mannitol did not affect viability. From this data, it can be inferred that SICD is exacerbated by readily metabolized carbon sources.

### Intermediates of sugar metabolism induce cell death of *S. cerevisiae* but do not support growth

We suspected that carbon metabolism leads to the production of a common metabolite that acts as a signal for cell death. To investigate this hypothesis, we tested if intermediates of carbon metabolism, specifically 4% pyruvate, 4% glycerol, 4% succinate, and 4% ethanol, can support growth and induce cell death in S288c-PDA. Growth was determined after 48 hours of incubation. Glycerol was the only intermediate that supported growth, although poorly, when compared to glucose and fructose. Supplementation with pyruvate and succinate resulted in a minimal increase in growth, while ethanol did not result in growth (Fig. 5A). However, it is noteworthy that growth in ethanol initiated beyond 72 hours.

We then determined the effect of these intermediates on cell viability. In comparison to glucose and fructose, we found that viability remained unchanged over 72 hours in glycerol; however, a 2-log, 3-log, and 4-log reduction was observed with pyruvate, succinate, and ethanol, respectively (Fig. 5C). It was previously reported that 5% ethanol is not toxic to *S. cerevisiae* BY4741 and W303 (19); therefore, the decline in survival seen with ethanol is perhaps not due to ethanol toxicity. Succinate reduced viability to a similar extent as glucose at each sampling interval, suggesting that it may have an important role in cell death signaling.

### SICD in *Candida* spp. is not dependent on sugar metabolism

**Fig 6:**
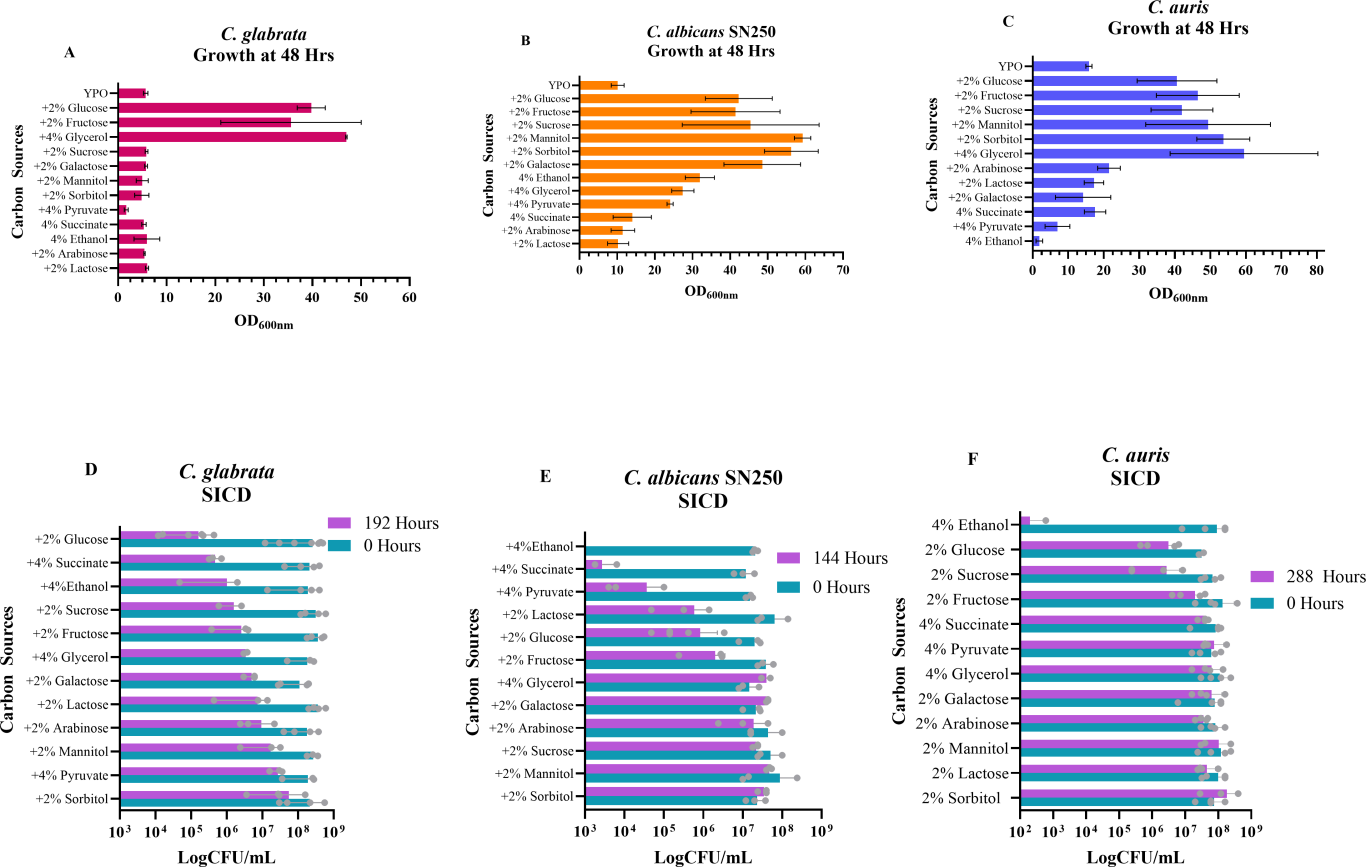
The effect of other carbon sources on survival of *Candida* spp. Metabolized carbon sources were determined for *C. glabrata* (**A**), *C. albicans* SN250 (**B**), and *C. auris* (**C**) by inoculating them in YP-only supplemented with 2% of respective sugars or 4% of the intermediates. The OD_600_ was measured at 48 hours to determine if growth is supported. SICD assays were done with the same concentration of carbon sources in water, and viability was determined before incubation and 192 hours for *C. glabrata* (**D**), 144 hours for *C. albicans* SN250 (**E**), and 288 hours for *C. auris* (**F**). The data presented in panels A to C are mean ± standard deviation, and the data presented in panels C to F are mean with range. Each dot represents the data collected from independent replicates.

We then asked if SICD in *Candida* spp. would have similarity to our findings with *S. cerevisiae*. Using the carbon sources and intermediates previously mentioned, we identified which of those are metabolized and non-metabolized by *C. glabrata*, *C. albicans* SN250, and *C. auris* and then assessed their effect on cell viability (Fig. 6). As shown in Fig. 6A, growth of *C. glabrata* was strongly supported by glucose, fructose, and glycerol, but not the other tested carbon sources. Glucose and succinate resulted in a 3-log reduction of viability, while ethanol, sucrose, fructose, glycerol, and galactose resulted in a 2-log reduction. Lactose, arabinose, and mannitol resulted in a 1-log reduction in viability, while sorbitol and pyruvate produced minimal changes in viability. In summary, the greatest decline in viability was caused by glucose, followed by succinate (Fig. 6D).

As shown in Fig. 6B, the growth of *C. albicans* SN250 was supported by all the tested carbon sources, although poorly with lactose, arabinose, and succinate, when compared to YP-only. Incubation in ethanol resulted in 0 CFU/mL by the first sample collected at 48 hours. Succinate resulted in the strongest cell death response, with a 3-log decline in viability, while pyruvate, glucose, lactose, and fructose resulted in about a 2-log reduction by 144 hours (Fig. 6E). Viability in glycerol, galactose, arabinose, sucrose, mannitol, and sorbitol remained unchanged for 144 hours.

Growth of *C. auris* was supported by glucose, fructose, sucrose, mannitol, sorbitol, and glycerol. Growth in arabinose, lactose, and succinate was poor, while there was no growth in ethanol, pyruvate, and galactose (Fig. 6C). Glucose and sucrose were the only carbon sources that reduced viability by nearly 2 logs by 288 hours. All other carbon sources did not result in a significant reduction in viability. In multiple trials, viability of *C. auris* declined to 0 CFU/mL by 48 hours in 4% ethanol (Fig. 6F).

In summary, growth of *C. albicans* and *C. auris* is supported by an array of carbon sources, but cell death is induced by a few. Conversely, *C. glabrata* grows on a limited number of our selected carbon sources, but cell death can be induced by many of them.

### Glucose incubation causes a decline in extracellular pH by *Candida* spp.

**Fig 7:**
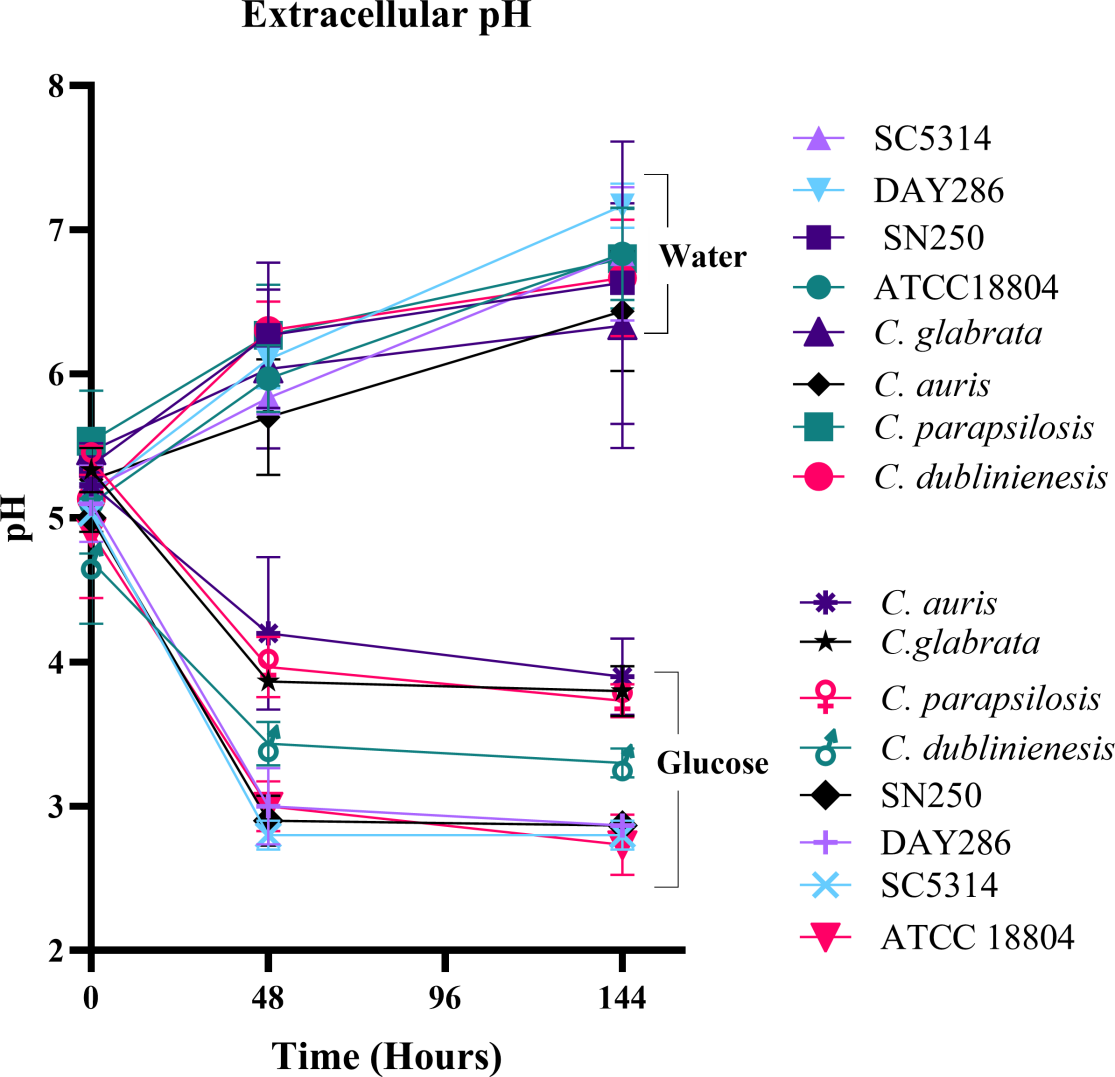
Change in pH upon incubation in glucose. Stationary-phase cells of *Candida* spp. were inoculated in either water or 2% glucose solutions, incubated at 37°C, and pH of the cultures was monitored. The data presented are mean ± standard deviation.

When *S. cerevisiae* is transferred to glucose-only solutions, there is a decline in extracellular pH (9). We hypothesized that this may also occur in other strains undergoing SICD. Stationary-phase cells of *C. albicans*, *C. dublinienesis*, *C. parapsilosis,* and *C. glabrata* were inoculated in either glucose- or water-only, and pH was assessed over time. As shown in Fig. 7, in water, the pH of all strains increased; however, in glucose, the pH declined and remained low throughout the incubation period. Interestingly, even though *C. dubliniensis* and *C. parapsilosis* are less sensitive to SICD, pH decreased in the presence of glucose. These data suggest that a decline in pH upon exposure to glucose is a universal response in yeast and may not be associated with the cell death response.

## DISCUSSION

Our study has revealed that SICD is prevalent in *S. cerevisiae* and, for the first time, *Candida*. All tested strains of *S. cerevisiae* and *Candida*, except for *C. dubliniensis*, exhibited SICD at 37°C. We determined that the rate of SICD in *S. cerevisiae* is dependent on the growth phase of cells (Fig. 1A and B), whereby exponential-phase cells undergo SICD more rapidly than stationary-phase cells in the first 24 hours. This is consistent with previous studies detecting signs of SICD within 15 minutes when exponential-phase cells were transferred to a glucose-only solution (8), while SICD in stationary-phase cells was reported to occur days later (1–3). This slower rate of cell death seen with stationary-phase cells is perhaps owing to changes that occur during the stationary phase of growth, such as decreased growth rate and a thickening of the cell wall, thereby delaying cell death (20–23) or the induction of autophagy in stationary phase, which is known to extend the lifespan of yeast (24, 25).

It was also found that stationary-phase cells of *S. cerevisiae* BY4741 are more tolerant to osmotic stress than exponential-phase cells (Fig. 1B). This is supported by previous studies showing that at 37°C, there is a decline in survival of this strain when transferred to water-only. This decline is not seen at 30°C, suggesting that temperature is playing a role in the response to osmotic stress (16).

We also observed that temperature is an important factor for SICD in *S. cerevisiae*. Reports in the literature highlighted that SICD is enhanced at 37°C (5), but can occur at 30°C (1, 2) as well as 25°C (1) and 20°C (4). However, those studies reported cell death as percentage death or percentage survival, which may potentially skew the data since a small difference in CFU may appear as a large change in percentage. In contrast, we conducted SICD testing in *S. cerevisiae* within a 72-hour period, and the data is presented as log CFU/mL to account for the exponential nature of growth seen in yeast. Our study revealed that within 72 hours, SICD is only detected at 37°C and not 30°C. Additionally, at 37°C, all tested strains survived in water. These results were reproduced in three strains of *S. cerevisiae* (Fig. 3) and four strains of *C. albicans* (Fig. 4). Our data suggest that physiological temperature and lack of nutrients synergistically promote SICD; therefore, this pathway may be a potential target for therapeutics.

We also found, for the first time, SICD in *C. albicans,* in both stationary- and exponential-phase cells (Fig. 2A through C). We suspect that this phenotype was missed in *C. albicans* because previous studies were done at 30°C (17), while we conducted our study at both 30°C and 37°C. Our finding of SICD in multiple strains of *C. albicans* at 37°C (Fig. 2A through C) and the absence of SICD at 30°C (Fig. 4) further support our hypothesis that temperature is a critical contributing factor of SICD. Additionally, SICD was also detected in both stationary- and exponential-phase cells of *C. glabrata* (Fig. 2D and E), exponential-phase cells of *C. parapsilosis*, and stationary-phase cells of *C. auris* (Fig. 2F).

Our data suggest that SICD was not detected in *C. dubliniensis* (Fig. 2D and E) in 2% glucose at 37°C because of its increased sensitivity to osmotic stress, which is consistent with a previous report (18). As seen in Fig. 2D, when sorbitol was supplemented, there was no change in viability. Additionally, *C. dubliniensis* is phylogenetically related to *C. albicans*, with 96.3% of genes sharing >80% identity, and is often quite difficult to distinguish from *C. albicans* phenotypically (26). Genomic studies have shown that *C. dubliniensis* went through reductive evolution and has become more sensitive to environmental stressors than *C. albicans*. There is reported divergent evolution between *C. albicans* and *C. dubliniensis* since speciation, where *C. dubliniensis* has lost genes important for pathogenicity, which may explain its poor ability to thrive as a commensal and opportunistic pathogen like *C. albicans* (18, 27). It is possible that *C. dubliniensis* also lost the ability to undergo SICD in this process of reductive evolution and may serve as an important genomic tool in elucidating the genetic and molecular mechanisms of SICD.

Additionally, we found that cell death was induced by more carbon sources in *S. cerevisiae* and *C. glabrata* (Fig. 5B, C , and 6D) than in *C. albicans* and *C. auris* (Fig. 6E and F). In accordance with the literature (28, 29), *S. cerevisiae* and *C. glabrata* appeared to be more metabolically specialized, using a smaller number of the tested sugars (Fig. 5A and 6A). On the other hand, *C. albicans* and *C. auris* were able to grow on a range of different sugars (Fig. 6B and D), highlighting their metabolic flexibility (29, 30). This inverse relationship between metabolic flexibility and cell death suggests that metabolic flexibility offers protection against cell death, perhaps by allowing for adaptation to alternative energy sources or the utilization of reserves to support survival.

Additionally, we found succinate to be a potent inducer of cell death in *S. cerevisiae* (Fig. 5C), *C. glabrata* (Fig. 6D), and *C. albicans* (Fig. 6E). It is yet to be determined if succinate is produced when cells are transferred to glucose-only solutions and whether that is directly associated with SICD. Determining the intracellular succinate levels and monitoring gene expression of TCA cycle genes, such as *LSC1* and *LSC2* (succinyl CoA-synthase) (31) when cells are exposed to glucose-only solutions, may provide important insights into the role of succinate in SICD.

**Fig 8:**
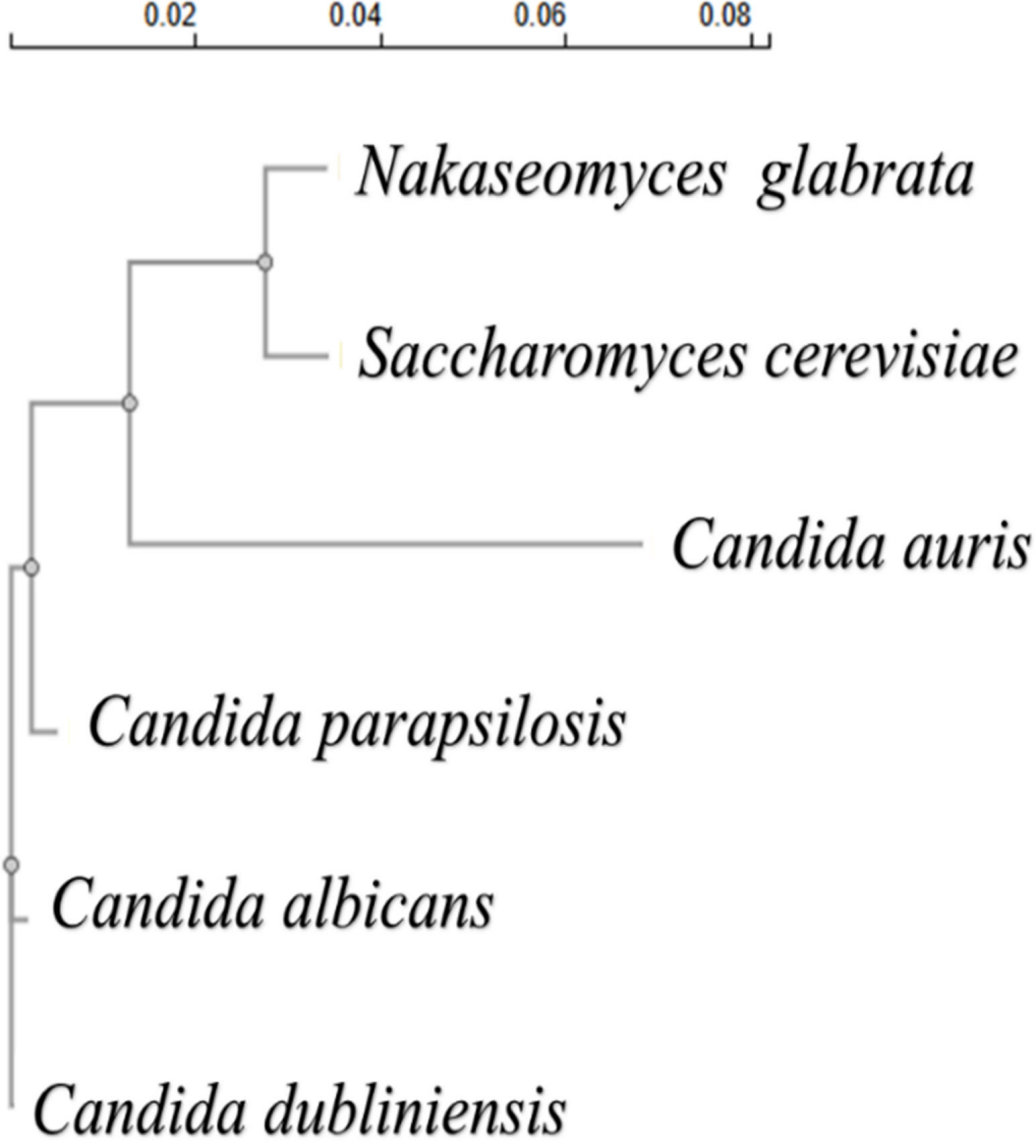
Evolutionary connection of the yeast tested in this study. Phylogenetic tree constructed based on 18s rRNA.

The similarity in sugar metabolism and SICD between *S. cerevisiae* and *C. glabrata/N. glabratus*, *C. auris*, and *C. albicans* is perhaps owing to its phylogenetic relationship (Fig. 8). Like *S. cerevisiae*, *C. glabrata* is a post-genome duplication (WGD) yeast that evolved to survive in different environments. The evolutionary relationship of the tested strains is shown in the phylogenetic tree constructed in Fig. 8. The presence of SICD in *C. albicans*, a pre-WGD yeast, suggests that SICD is an ancient phenotype. Our study showed that SICD is enhanced in the post-WGD yeasts *S. cerevisiae* and *C. glabrata*, leading to the idea that SICD is perhaps selected for through WGD, like the Crabtree effect (32–34). It should be noted that *C. auris* appears to be resistant to SICD by many of the tested sugars, supporting prior studies on the use of metabolic flexibility to promote its pathogenicity (30, 35).

Similar types of cell death are induced by high glucose in mammalian cells, especially in cases of hyperglycemia and diabetes (36–38). Several studies have shown that high glucose-induced cell death in mammalian cells shares hallmarks of SICD observed in *S. cerevisiae* (36, 39–41), thereby suggesting that the phenomenon of SICD may also be conserved in higher eukaryotes.

Previous studies have suggested that SICD occurs due to the disruption of the Crabtree effect (11, 42). Since Crabtree-negative yeast *C. albicans* and *C. auris* were found to be less sensitive to SICD (Fig. 6E and F), the perturbation of the Crabtree effect is unlikely to be solely responsible for SICD. Additionally, incubation of *S. cerevisiae* in glucose-only solutions results in a rapid decline in pH due to activation of H^+^-ATPases and release of carboxylic acids (6, 9, 10). Interestingly, we observed the same response when *Candida* spp. was introduced to glucose-only solutions (Fig. 7). This decline in pH was also seen in *C. dublinienesis*, which is SICD-negative under the conditions used in this study*,* suggesting that cell death may not be directly linked to a decline in extracellular pH.

### Conclusion

Together, our data suggest that SICD may be a fundamental and evolutionarily conserved phenotype in yeast and is possibly enhanced in post-WGD yeast. We also provide strong evidence of physiological temperature being a critical factor in the induction of SICD and a universal decline in pH upon incubation of yeast in glucose-only solutions.

## References

[1] Granot D, Snyder M. 1991. Glucose induces cAMP-independent growth-related changes in stationary-phase cells of Saccharomyces cerevisiae. Proc Natl Acad Sci USA 88:5724–5728. doi:10.1073/pnas.88.13.5724 1648229 PMC51950

[2] Granot D, Snyder M. 1993. Carbon source induces growth of stationary phase yeast cells, independent of carbon source metabolism. Yeast 9:465–479. doi:10.1002/yea.320090503 8322510

[3] Granot D, Levine A, Dor-Hefetz E. 2003. Sugar-induced apoptosis in yeast cells. FEMS Yeast Res 4:7–13. doi:10.1016/S1567-1356(03)00154-5 14554192

[4] Yoshimoto H, Ohuchi R, Ikado K, Yoshida S, Minato T, Ishiguro T, Mizutani S, Kobayashi O. 2009. Sugar induces death of the bottom fermenting yeast Saccharomyces pastorianus. J Biosci Bioeng 108:60–62. doi:10.1016/j.jbiosc.2008.12.022 19577194

[5] Granot D, Dai N. 1997. Sugar induced cell death in yeast is dependent on the rate of sugar phosphorylation as determined by Arabidopsis thaliana hexokinase. Cell Death Differ 4:555–559. doi:10.1038/sj.cdd.4400280 14555968

[6] Hoeberichts FA, Perez-Valle J, Montesinos C, Mulet JM, Planes MD, Hueso G, Yenush L, Sharma SC, Serrano R. 2010. The role of K(+) and H(+) transport systems during glucose- and H(2)O(2)-induced cell death in Saccharomyces cerevisiae. Yeast 27:713–725. doi:10.1002/yea.1767 20213854

[7] Carmona-Gutierrez D, Bauer MA, Zimmermann A, Aguilera A, Austriaco N, Ayscough K, Balzan R, Bar-Nun S, Barrientos A, Belenky P, et al.. 2018. Guidelines and recommendations on yeast cell death nomenclature. Microb Cell 5:4–31. doi:10.15698/mic2018.01.607 29354647 PMC5772036

[8] Valiakhmetov AY, Kuchin AV, Suzina NE, Zvonarev AN, Shepelyakovskaya AO. 2019. Glucose causes primary necrosis in exponentially grown yeast Saccharomyces cerevisiae. FEMS Yeast Res 19:foz019. doi:10.1093/femsyr/foz019 30785621

[9] Bidiuk VA, Alexandrov AI, Valiakhmetov AY. 2021. Extracellular pH and high concentration of potassium regulate the primary necrosis in the yeast Saccharomyces cerevisiae. Arch Microbiol 204:35. doi:10.1007/s00203-021-02708-6 34927223

[10] Avtukh A, Baskunov B, Keshelava V, Valiakhmetov A. 2023. Sugar-induced cell death in the yeast S. cerevisiae is accompanied by the release of octanoic acid, which does not originate from the fatty acid synthesis type II mitochondrial system. Appl Microbiol 3:722–734. doi:10.3390/applmicrobiol3030050

[11] Lee YJ, Burlet E, Galiano F, Circu ML, Aw TY, Williams BJ, Witt SN. 2011. Phosphate and succinate use different mechanisms to inhibit sugar-induced cell death in yeast: insight into the Crabtree effect. J Biol Chem 286:20267–20274. doi:10.1074/jbc.M110.209379 21515692 PMC3121461

[12] Ahn S-H, Cheung WL, Hsu J-Y, Diaz RL, Smith MM, Allis CD. 2005. Sterile 20 kinase phosphorylates histone H2B at serine 10 during hydrogen peroxide-induced apoptosis in S. cerevisiae. Cell 120:25–36. doi:10.1016/j.cell.2004.11.016 15652479

[13] Díaz-Ruiz R, Avéret N, Araiza D, Pinson B, Uribe-Carvajal S, Devin A, Rigoulet M. 2008. Mitochondrial oxidative phosphorylation is regulated by fructose 1,6-bisphosphate. A possible role in Crabtree effect induction? J Biol Chem 283:26948–26955. doi:10.1074/jbc.M800408200 18682403

[14] Du H, Liang Y. 2006. Saccharomyces cerevisiae ste20 mutant showing resistance to glucose-induced cell death. Yi Chuan Xue Bao 33:664–668. doi:10.1016/S0379-4172(06)60097-8 16875325

[15] Lee YJ, Burlet E, Wang S, Xu B, Huang S, Galiano FJ, Witt SN. 2011. Triclabendazole protects yeast and mammalian cells from oxidative stress: Identification of a potential neuroprotective compound. Biochem Biophys Res Commun 414:205–208. doi:10.1016/j.bbrc.2011.09.057 21946065 PMC3195900

[16] Dušková M, Cmunt D, Papoušková K, Masaryk J, Sychrová H. 2021. Minority potassium-uptake system Trk2 has a crucial role in yeast survival of glucose-induced cell death. Microbiology (Reading) 167. doi:10.1099/mic.0.001065 34170815

[17] Du H, Guan G, Li X, Gulati M, Tao L, Cao C, Johnson AD, Nobile CJ, Huang G. 2015. N-acetylglucosamine-induced cell death in Candida albicans and its implications for adaptive mechanisms of nutrient sensing in yeasts. mBio 6:e01376-15. doi:10.1128/mBio.01376-15 26350972 PMC4600118

[18] Enjalbert B, Moran GP, Vaughan C, Yeomans T, Maccallum DM, Quinn J, Coleman DC, Brown AJP, Sullivan DJ. 2009. Genome-wide gene expression profiling and a forward genetic screen show that differential expression of the sodium ion transporter Ena21 contributes to the differential tolerance of Candida albicans and Candida dubliniensis to osmotic stress. Mol Microbiol 72:216–228. doi:10.1111/j.1365-2958.2009.06640.x 19239621

[19] Kubota S, Takeo I, Kume K, Kanai M, Shitamukai A, Mizunuma M, Miyakawa T, Shimoi H, Iefuji H, Hirata D. 2004. Effect of ethanol on cell growth of budding yeast: genes that are important for cell growth in the presence of ethanol. Biosci Biotechnol Biochem 68:968–972. doi:10.1271/bbb.68.968 15118337

[20] Herman PK. 2002. Stationary phase in yeast. Curr Opin Microbiol 5:602–607. doi:10.1016/s1369-5274(02)00377-6 12457705

[21] Chen Q, Ding Q, Keller JN. 2005. The stationary phase model of aging in yeast for the study of oxidative stress and age-related neurodegeneration. Biogerontology 6:1–13. doi:10.1007/s10522-004-7379-6 15834659

[22] Broach JR. 2012. Nutritional control of growth and development in yeast. Genetics 192:73–105. doi:10.1534/genetics.111.135731 22964838 PMC3430547

[23] Smith AE, Zhang Z, Thomas CR, Moxham KE, Middelberg APJ. 2000. The mechanical properties of Saccharomyces cerevisiae. Proc Natl Acad Sci USA 97:9871–9874. doi:10.1073/pnas.97.18.9871 10963659 PMC27610

[24] Adachi A, Koizumi M, Ohsumi Y. 2017. Autophagy induction under carbon starvation conditions is negatively regulated by carbon catabolite repression. J Biol Chem 292:19905–19918. doi:10.1074/jbc.M117.817510 29042435 PMC5712628

[25] Iwama R, Ohsumi Y. 2019. Analysis of autophagy activated during changes in carbon source availability in yeast cells. J Biol Chem 294:5590–5603. doi:10.1074/jbc.RA118.005698 30755486 PMC6462502

[26] Moran GP, Coleman DC, Sullivan DJ. 2012. Candida albicans versus Candida dubliniensis: why is C. albicans more pathogenic? Int J Microbiol 2012:205921. doi:10.1155/2012/205921 21904553 PMC3166774

[27] Jackson AP, Gamble JA, Yeomans T, Moran GP, Saunders D, Harris D, Aslett M, Barrell JF, Butler G, Citiulo F, et al.. 2009. Comparative genomics of the fungal pathogens Candida dubliniensis and Candida albicans. Genome Res 19:2231–2244. doi:10.1101/gr.097501.109 19745113 PMC2792176

[28] Brunke S, Hube B. 2013. Two unlike cousins: Candida albicans and C. glabrata infection strategies. Cell Microbiol 15:701–708. doi:10.1111/cmi.12091 23253282 PMC3654559

[29] Van Ende M, Wijnants S, Van Dijck P. 2019. Sugar sensing and signaling in Candida albicans and Candida glabrata. Front Microbiol 10:10. doi:10.3389/fmicb.2019.00099 30761119 PMC6363656

[30] Weerasinghe H, Simm C, Djajawi TM, Tedja I, Lo TL, Simpson DS, Shasha D, Mizrahi N, Olivier FAB, Speir M, Lawlor KE, Ben-Ami R, Traven A. 2023. Candida auris uses metabolic strategies to escape and kill macrophages while avoiding robust activation of the NLRP3 inflammasome response. Cell Rep 42:112522. doi:10.1016/j.celrep.2023.112522 37204928

[31] Przybyla-Zawislak B, Dennis RA, Zakharkin SO, McCammon MT. 1998. Genes of succinyl-CoA ligase from Saccharomyces cerevisiae. Eur J Biochem 258:736–743. doi:10.1046/j.1432-1327.1998.2580736.x 9874242

[32] Rozpędowska E, Galafassi S, Johansson L, Hagman A, Piškur J, Compagno C. 2011. Candida albicans--a pre-whole genome duplication yeast--is predominantly aerobic and a poor ethanol producer. FEMS Yeast Res 11:285–291. doi:10.1111/j.1567-1364.2010.00715.x 21205163

[33] Malina C, Yu R, Björkeroth J, Kerkhoven EJ, Nielsen J. 2021. Adaptations in metabolism and protein translation give rise to the Crabtree effect in yeast. Proc Natl Acad Sci USA 118:e2112836118. doi:10.1073/pnas.2112836118 34903663 PMC8713813

[34] Roetzer A, Gabaldón T, Schüller C. 2011. From Saccharomyces cerevisiae to Candida glabratain a few easy steps: important adaptations for an opportunistic pathogen. FEMS Microbiol Lett 314:1–9. doi:10.1111/j.1574-6968.2010.02102.x 20846362 PMC3015064

[35] Simm C, Weerasinghe H, Thomas DR, Harrison PF, Newton HJ, Beilharz TH, Traven A. 2022. Disruption of iron homeostasis and mitochondrial metabolism are promising targets to inhibit Candida auris. Microbiol Spectr 10:e00100-22. doi:10.1128/spectrum.00100-22 35412372 PMC9045333

[36] Allen DA, Yaqoob MM, Harwood SM. 2005. Mechanisms of high glucose-induced apoptosis and its relationship to diabetic complications. J Nutr Biochem 16:705–713. doi:10.1016/j.jnutbio.2005.06.007 16169208

[37] Ceriello A, Russo P de, Amstad P, Cerutti P. 1996. High glucose induces antioxidant enzymes in human endothelial cells in culture. Evidence linking hyperglycemia and oxidative stress. Diabetes 45:471–477. doi:10.2337/diabetes.45.4.471 8603769

[38] Lorenzi M, Cagliero E, Toledo S. 1985. Glucose toxicity for human endothelial cells in culture. Delayed replication, disturbed cell cycle, and accelerated death. Diabetes 34:621–627. doi:10.2337/diab.34.7.621 3924693

[39] Sheu ML, Ho FM, Yang RS, Chao KF, Lin WW, Lin-Shiau SY, Liu S-H. 2005. High glucose induces human endothelial cell apoptosis through a phosphoinositide 3-kinase-regulated cyclooxygenase-2 pathway. Arterioscler Thromb Vasc Biol 25:539–545. doi:10.1161/01.ATV.0000155462.24263.e4 15653566

[40] Curcio F, Ceriello A. 1992. Decreased cultured endothelial cell proliferation in high glucose medium is reversed by antioxidants: new insights on the pathophysiological mechanisms of diabetic vascular complications. In Vitro Cell Dev Biol 28A:787–790. doi:10.1007/BF02631069 1483970

[41] Parbhudayal R, Cheng H-P. 2025. Exploring sugar-induced cell death in yeast: implications for diabetes and cancer research. Front Cell Death 4:4. doi:10.3389/fceld.2025.1470093 PMC12539669 41127177

[42] de Alteriis E, Cartenì F, Parascandola P, Serpa J, Mazzoleni S. 2018. Revisiting the Crabtree/Warburg effect in a dynamic perspective: a fitness advantage against sugar-induced cell death. Cell Cycle 17:688–701. doi:10.1080/15384101.2018.1442622 29509056 PMC5969562

